# Assessment of a Peer Physician Coaching Partnership Between a Designated Cancer Center Genetics Service and a Community Cancer Network Hospital

**DOI:** 10.1001/jamanetworkopen.2023.1723

**Published:** 2023-03-06

**Authors:** Lauren G. Santos, Tatyana Buzdnitskaya, Bradley A. Rolf, William Souza, Mark Sienko, Jose Alberto Ruiz-Bonilla, Binay Shah, Patrick Jewell, Lindsay Jensen, Martha Horike-Pyne, Jo Ann Elrod, Jennie Crews, Mercy Laurino, Kevin Austin Weeks, Marianne E. Dubard-Gault

**Affiliations:** 1 Department of Medicine, University of Washington, Seattle; 2 Clinical Cancer Genetics Service, Fred Hutchinson Cancer Center, Seattle, Washington; 3 Olympic Medical Cancer Center, Sequim, Washington

## Abstract

**Background:**

Patients with cancer seen in rural and underserved areas disproportionately face barriers to access genetic services. Genetic testing is critical to inform treatment decisions, for early detection of another cancer, and to identify at-risk family members who may benefit from screening and prevention.

**Objective:**

To examine medical oncologists’ genetic testing ordering trends for patients with cancer.

**Design, Setting, and Participants:**

This prospective quality improvement study was performed in 2 phases over 6 months between August 1, 2020, and January 31, 2021, at a community network hospital. Phase 1 focused on observation of clinic processes. Phase 2 incorporated peer coaching from cancer genetics experts for medical oncologists at the community network hospital. The follow-up period lasted 9 months.

**Main Outcomes and Measures:**

The number of genetic tests ordered was compared between phases.

**Results:**

The study included 634 patients (mean [SD] age, 71.0 [10.8] years [range, 39-90 years]; 409 women [64.5%]; 585 White [92.3%]); 353 (55.7%) had breast cancer, 184 (29.0%) had prostate cancer, and 218 (34.4%) had a family history of cancer. Of the 634 patients with cancer, 29 of 415 (7.0%) received genetic testing in phase 1, and 25 of 219 (11.4%) received genetic testing in phase 2. Of the 29 patients who received testing in phase 1, 20 (69.0%) had germline genetic testing; 23 of 25 patients (92.0%) had germline genetic testing in phase 2. Uptake of germline genetic testing increased by 23.0% between phases, but the difference was not statistically significant (*P* = .06). Uptake of germline genetic testing was highest among patients with pancreatic cancer (4 of 19 [21.1%]) and ovarian cancer (6 of 35 [17.1%]); the National Comprehensive Cancer Network (NCCN) recommends offering genetic testing to all patients with pancreatic cancer and ovarian cancer.

**Conclusions and Relevance:**

This study suggests that peer coaching from cancer genetics experts was associated with an increase in ordering of genetic testing by medical oncologists. Efforts made to (1) standardize gathering of personal and family history of cancer, (2) review biomarker data suggestive of a hereditary cancer syndrome, (3) facilitate ordering tumor and/or germline genetic testing every time NCCN criteria are met, (4) encourage data sharing between institutions, and (5) advocate for universal coverage for genetic testing may help realize the benefits associated with precision oncology for patients and their families seeking care at community cancer centers.

## Key Points

**Question:**

Does peer coaching of community oncologists increase ordering of germline genetic testing when patients with active cancer meet criteria for such testing?

**Findings:**

In this quality improvement study with 634 patients, peer coaching was associated with increased ordering and uptake of genetic testing for patients with active cancer. Further support is needed to deliver genetic testing at diagnosis of cancer every time patients meet criteria, as only 1 in 5 patients with pancreatic cancer received germline genetic testing in 2020.

**Meaning:**

In this study, peer coaching was insufficient in delivering germline genetic testing to patients with active cancer; these findings suggest that having genetic counseling support would enable community oncologists to order genetic testing every time criteria are met.

## Teaser

This quality improvement study examines medical oncologists’ genetic testing ordering trends for patients with cancer.

## Introduction

Patients seen in rural and underserved areas often face health disparities, encounter barriers to access health care services, and have worse outcomes compared with patients in urban communities.^1^ In the context of precision oncology, patients with cancer seen in rural health facilities are less likely to receive the benefits associated with tumor profiling.^2^ Consequently, they may lack awareness that inherited genetic traits can guide cancer treatment or that a genetics evaluation can help with screening as well as with early detection for them and at-risk relatives.^3,4^ Even when patients with cancer are referred for counseling and testing, they face obstacles in accessing testing and follow-up services for a hereditary cancer syndrome (HCS).^5,6,7^

Identification of an HCS may allow for individualization of targeted therapies, prediction of response, risk stratification, tailored screening, early detection, and risk reduction interventions.^8,9,10,11^ Cancer genetic services also empower relatives of patients with an HCS to be proactive in their preventive health care. Relatives at risk of cancer meet criteria for high-risk screening and early detection protocols and may also benefit from risk reduction interventions when possible.^12,13,14^

This pilot project was the first collaboration between the Fred Hutchinson Cancer Center (FHCC) cancer genetics service in the greater Seattle, Washington, area and one of its affiliated network regional community-based cancer centers, the Olympic Medical Cancer Center (OMCC), located on the Olympic peninsula in Washington state. Before April 1, 2022, the FHCC was formerly known as the Seattle Cancer Care Alliance and the Fred Hutchinson Cancer Research Center. Our goal was to address the gap of access to genetic testing services when patients meet National Comprehensive Cancer Network (NCCN) criteria through a peer coaching intervention and ad hoc consultations from the FHCC cancer genetics service.

## Methods

This quality improvement study was performed in 2 phases over 6 months between August 1, 2020, and January 31, 2021. We expected to have 250 new patients given that there were 246 patients with a new diagnosis of breast, colon, ovarian, pancreatic, or prostate cancer at the OMCC in 2019. Patient informed consent was waived because the study was deemed minimal risk. All 6 community oncologists consented to participating in coaching. We received an exemption from the University of Washington institutional review board for the quality improvement part of the study because the study was deemed minimal risk and received approval for peer coaching of oncologists. This study followed the Standards for Quality Improvement Reporting Excellence (SQUIRE) reporting guideline for quality improvement studies.

We identified our cohort by selecting patients with a new or active diagnosis of breast, ovarian, colon, pancreatic, and/or prostate cancer who were seen at the OMCC. Demographic characteristics, clinical history, family history data, and pathology reports were collected through electronic health record (EHR) system–generated reports or through medical record review when available. Most patients seen within phase 1 and phase 2 had an overlap of visits, making it difficult to differentiate when genetic testing was discussed and/or ordered within each distinct phase. Our initial EHR data collection included 1349 patient encounters in phase 1 and 1124 patient encounters in phase 2 (Figure). We separated patient visits based on the first encounter date for cancer care as either before or after August 1, 2020. Given that patients were seen for multiple visits over their first year, we further separated initial consultations vs follow-up visits. We then assigned patients seen for an initial consultation to phase 1 or phase 2 using the date when testing was ordered or, when no testing was ordered, the consultation date. Patients with cancer diagnosed before August 1, 2020, who had only follow-up visits in both phase 1 and phase 2, as well as genetic testing, were assigned based on the date their genetic test was ordered. Patients who had only follow-up visits within the project timeline and for whom no genetic testing was ordered were categorized as phase 1 patients. The number of patients seen within phase 1 and phase 2 were affected by the social distancing mandates related to the COVID-19 pandemic (Figure).^15^

**Figure.**
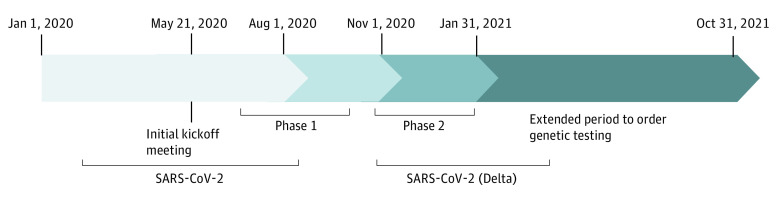
Timeline of SARS-CoV-2 Infection Waves in the United States Using Data From the World Health Organization and the Centers for Disease Control and Prevention

The study team included an oncogeneticist (M.E.D.-G.), a certified genetic counselor (B.A.R.), a principal study coordinator (L.G.S.), study site coordinators (T.B. and W.S.), and oncology clinicians (M.S., J.A.R.-B., B.S., P.J., L.J., and K.A.W.). The study team collaborated with Invitae, a College of American Pathologists–accredited and Clinical Laboratory Improvement Amendments of 1988–certified clinical diagnostic laboratory, that provided in-service education on the online ordering portal before the study started. Invitae was selected as the testing laboratory due to its shorter turnaround time, assistance with prior authorization, and no-cost family testing program when a patient’s genetic testing results were positive. Invitae genetic testing was covered by study funds regardless of whether peer coaching was provided to OMCC oncologists. All patients were offered the same hereditary cancer panel of 47 genes because it provided a comprehensive test for the main actionable HCS. The study team used a modified version of the NCCN guidelines for genetic testing that stemmed from the 2020-2021 NCCN guidelines^16^ (Table 1) because the OMCC clinical support staff were not trained in cancer genetics and were pressed for time when gathering medical records and family history. For patients with a complicated history, the OMCC team offered a referral to the FHCC genetics service.

**Table 1.** Simplified NCCN Guidelines for Germline Genetic Testing by Disease Group

Disease group	Simplified NCCN guidelines for germline genetic testing
Breast	Every breast cancer diagnosis for a patient <60 y
	Any breast cancer diagnosis for a patient with a family history of any cancer
Colon	Every colon cancer diagnosis for a patient <50 y
	Any colon cancer diagnosis for a patient <70 y with a family history of any cancer
	Somatic variants in the mismatch DNA repair or homologous recombination DNA repair pathway
Ovarian	Every patient with an ovarian cancer diagnosis or family history of ovarian cancer
Pancreatic	Every patient with a pancreatic cancer diagnosis or family history of pancreatic cancer
Prostate	Every prostate cancer diagnosis for patients <70 y with either a family history or high-risk features, such as a Gleason score ≥4 + 4 or positive lymph nodes
	Every patient with metastatic prostate cancer
	Somatic variants in the homologous recombination or mismatch DNA repair pathway

Abbreviation: NCCN, National Comprehensive Cancer Network.

Phase 1 focused on observing the clinical processes that the OMCC used to identify patients meeting modified NCCN guidelines. We reviewed oncology visit details and tracked the use of genetic testing. We focused on the cancer diagnoses listed to have a large enough subset in each group and because genetic testing guidelines have been available for more than a decade. Phase 2 included a peer coaching intervention for OMCC oncologists in counseling patients and ordering genetic testing for cases they brought forward at bimonthly virtual meetings. The study team reviewed deidentified patient information prior to or after clinic visits. The OMCC oncologists would then discuss the importance of hereditary cancer testing with eligible patients and offered genetic testing through Invitae at their discretion. We documented the use of genetic testing between August 1, 2020, and January 31, 2021. At the end of each phase, we measured the number of patients with cancer who met NCCN criteria, the number of patients who received genetic testing locally, and the number of patients referred to the FHCC genetic service. The number of tests ordered was then compared between phase 1 and 2.

### Statistical Analysis

We examined trends based on sex, age, race and ethnicity, cancer diagnosis, and family history. We correlated clinical, cancer diagnosis, family history, and demographic variables with ordering of genetic testing in both phase 1 and phase 2. We recorded the number of patients who met NCCN testing criteria. We compared the proportion of patients who received testing between phases and the types of genetic testing received before and after the peer coaching intervention. Analysis was performed using Excel, version 16.0 pivot tables and functions (Microsoft Corp). We used 2-sided χ^2^ tests with a *P* value threshold of *P* < .05 to assess whether the peer coaching intervention resulted in a statistically significant increase in genetic testing uptake.

## Results

### Study Population

The patient cohort included 634 patients (mean [SD] age, 71.0 [10.8] years; median age, 73 years [range, 39-90 years]; 409 women [64.5%] and 255 men [35.5%]; 585 White [92.3%]): 415 patients in phase 1 and 219 patients in phase 2 (Table 2). In phase 1, there were 54 initial consultations for oncology care (13.0%) and 361 follow-up visits (87.0%); in phase 2, there were 65 initial consultations for oncology care (29.7%) and 155 follow-up visits (70.8%). Among both phases, there were 353 patients with breast cancer (55.7%), 184 (29.0%) with prostate cancer, 43 (6.8%) with colon cancer, 35 (5.5%) with ovarian cancer, and 19 (3.0%) with pancreatic cancer. Of the 634 patients, 67 (10.6%) had stage 0 or 1 disease, 47 (7.4%) had stage 2 disease, 37 (5.8%) had stage 3 disease, and the stage was unavailable to the study team for 458 patients (72.2%). Of 634 patients, 218 (34.4%) had a family history of cancer documented. Of 415 patients in phase 1, 100 (24.1%) met modified NCCN criteria for genetic testing; of 219 patients in phase 2, 48 (21.9%) met modified NCCN criteria for genetic testing. Of all patients, 12 (1.9%) died during the study.

**Table 2.** Patient Demographic and Clinical Characteristics by Phase

Characteristic	Patients, No. (%)	
	Phase 1 (n = 415)	Phase 2 (n = 219)
Sex		
Male	156 (37.6)	69 (31.5)
Female	259 (62.4)	150 (68.5)
Age, y		
Mean (SD)	72 (11.0)	70 (10.4)
Median (range)	73 (47-83)	70 (38-90)
Race		
American Indian or Alaska Native	5 (1.2)	NR
Asian	7 (1.7)	NR
Black or African American	0	NR
White	382 (92.0)	203 (92.7)
Other^a^	9 (2.2)	NR
Unknown or declined to answer	12 (2.9)	5 (2.3)
Cancer diagnosis		
Breast	213 (51.3)	140 (63.9)
Prostate	138 (33.3)	46 (21.0)
Colon	23 (5.5)	20 (9.1)
Ovarian	29 (7.0)	6 (2.7)
Pancreatic	12 (2.9)	7 (3.2)
Cancer stage		
0 or 1	44 (10.6)	23 (10.5)
2	33 (8.0)	14 (6.4)
3	27 (6.5)	10 (4.6)
4	21 (5.1)	NR
Unknown	290 (69.9)	168 (76.7)
Genetic test results		
Received testing	29 (7.0)	25 (11.4)
Pathogenic and likely pathogenic, No./total No. (%)	7/29 (24.1)	2/25 (8.0)
Negative, No./total No. (%)	13/29 (44.8)	14/25 (56.0)
VUS or inconclusive, No./total No. (%)	9/29 (31.0)	9/25 (36.0)
Did not receive testing	386 (93.0)	194 (88.6)
Family history of cancer		
Yes	142 (34.2)	76 (34.7)
No	34 (8.2)	18 (8.2)
Pedigree unavailable	239 (57.6)	125 (57.1)
Modified NCCN criteria		
Met	100 (24.1)	48 (21.9)
Not met or unsure	315 (75.9)	171 (78.1)

Abbreviations: NCCN, National Comprehensive Cancer Network; NR, not reported (numbers were too small); VUS, variant of uncertain significance.

^a^ Includes Pacific Islander, race other than listed, more than 1 race, and mixed race.

### Uptake of Genetic Testing

Overall, 105 of 148 patients (70.9%) who met modified NCCN criteria did not receive genetic testing; 29 of 415 patients (7.0%) received genetic testing in phase 1 and 25 of 219 patients (11.4%) received genetic testing in phase 2. Of the 29 patients who received testing in phase 1, 20 (69.0%) had germline genetic testing and 9 (31.0%) had somatic testing; 7 patients (24.1%) had a pathogenic variant, 13 (44.8%) had a negative result, and 9 (31.0%) had a variant of uncertain significance. Of the 25 patients who received genetic testing in phase 2, 23 (92.0%) had germline genetic testing and 2 (8.0%) had somatic testing; 2 patients (8.0%) had a pathogenic variant, 14 (56.0%) had a negative result, and 9 (36.0%) had a variant of uncertain significance. Uptake of germline genetic testing increased by 23.0% between phase 1 and phase 2, but the difference between observed and expected values did not reach statistical significance (*P* = .06). Uptake of genetic testing was lowest among patients with prostate cancer (6.5% [12 of 184]).

### Trends

A 3-generation family history of cancer at cancer diagnosis is critical to estimate the pretest likelihood of an HCS and guide the choice of targeted therapy with the highest chance of benefit. We analyzed the association between documentation of family history and uptake of germline genetic testing. The proportions of patients who had (1) a documented family history of cancer in the EHR, (2) documentation they had no family history of cancer in the EHR, and (3) an unknown family history of cancer (either not documented in the EHR or unavailable to the study team) were almost identical in both phases and did not increase with peer coaching intervention in phase 2 (57.6% [239 of 415] undocumented in phase 1 vs 57.1% [125 of 219] undocumented in phase 2) (Table 3).

**Table 3.** Documentation of Family History and Uptake of Germline and Somatic Testing by Phase

Phase	Patients, No. (%)	
	Yes	No
**Phase 1 (August 1 to October 31, 2020) (n = 415)**		
Family history	142 (34.2)	273 (65.8)
No family history	34 (8.2)	381 (91.8)
No information	239 (57.6)	176 (42.4)
Germline testing	20 (4.8)	395 (95.2)
Somatic testing	9 (2.2)	406 (97.8)
Total of all testing	29 (7.0)	386 (93.0)
**Phase 2 (November 1, 2020, to January 31, 2021) (n = 219)**		
Family history	76 (34.7)	143 (65.3)
No family history	18 (8.2)	201 (91.8)
No information	125 (57.1)	94 (42.9)
Germline testing	23 (10.5)	196 (89.5)
Somatic testing	2 (0.9)	217 (99.1)
Total of all testing	25 (11.4)	194 (88.6)

Peer coaching intervention appears to have helped bring the conversation about hereditary testing back to the forefront of patients’ care. Despite there being no observed increase in documentation of family history of cancer, there was an association between clinician awareness of updated guidelines and offering germline genetic testing to patients who received a diagnosis of cancer prior to the August 1, 2020, study start date. There was no significant difference in the distribution of cancer types between phases. We were not able to observe meaningful differences in uptake of genetic testing between ancestries or by cancer stage because of the small numbers of patients or missing data. Uptake of germline genetic testing was highest among patients with pancreatic cancer (4 of 19 [21.1%]) and ovarian cancer (6 of 35 [17.1%]). This uptake of germline genetic testing may be due to updated NCCN guidelines starting January 1, 2020, recommending that all patients with pancreatic cancer^14^ or with ovarian cancer^17^ be offered genetic testing. In addition, based on the 2020 Commission on Cancer standard 4.4,^18^ all Commission on Cancer–accredited cancer centers in the US were required to report on the number of genetic counseling referrals for all patients of a specific cancer group; the OMCC elected to focus on pancreatic cancer.

## Discussion

To our knowledge, this project was the first partnership of its kind between the FHCC cancer genetics service and an affiliate regional community-based cancer center. The peer coaching intervention was associated with a 23.0% increase in uptake of germline genetic testing for patients with cancer treated at the OMCC. Although the increase was not statistically significant, it represented a marked improvement in access to genetic testing for patients and relatives over a short study period. Germline genetic testing was documented for 21.1% of patients with pancreatic cancer and 17.1% of patients with ovarian cancer, when the NCCN recommended that all of these patients be offered genetic testing. Another key takeaway was that no genetic testing was documented for 70.9% of patients who met modified NCCN criteria. This finding highlights a need for continued partnership and a potential need to simplify testing guidelines.

It is possible that genetic testing occurred with another laboratory, before the study start date, or after the study ended. In certain cases, copies of genetic testing reports were unavailable because the study team did not have access to other laboratory portals or to the faxed results stored within the OMCC’s EHR. Uptake of genetic testing was lowest among patients with prostate cancer (6.5%). One explanation may be that our study overlapped with another local study by Cheng et al^19^ offering sponsored germline genetic testing to all men with metastatic prostate cancer in Washington state (GENTleMEN [Genetic Testing for Men with Metastatic Prostate Cancer] study). In addition, the complexity of the most recent NCCN guidelines for genetic testing for patients with prostate cancer may have added additional challenges to offering testing for this subset of patients.

Most health insurers require that molecular oncology or germline genetic testing have prior authorization, and this work falls onto an already busy clinical staff. Even when insurance approval is obtained, patients with cancer are not guaranteed coverage. The risk of being charged large out-of-pocket costs makes many patients wary to pursue genetic testing and to forego genetic testing altogether. According to the US Census, the median income for Sequim, Washington, in Clallam County between 2016 and 2020 was $39 509 compared with $97 985 in Seattle, Washington, in King County.^20^ The lower median income would likely pose additional financial challenges if genetic testing costs were not covered by insurance. Hence, the availability of no-cost germline genetic testing may have also helped with the uptake of testing for patients with cancer at the OMCC.

The median patient age at the OMCC was 73 years, whereas the typical age range of patients seen at the FHCC campus is between 60 and 64 years. As the study progressed, it became clear that even the modified NCCN guidelines we implemented (Table 1) were not as helpful to uncover an HCS. Older patients with an HCS most often presented with a common cancer and had limited information about their deceased relatives, making it difficult to differentiate them from peers without an HCS. Over the past decade, cancer geneticists have found a higher number of patients carrying an HCS than anticipated; 1 in 8 people at cancer diagnosis, agnostic of cancer type, have an HCS^21^ and 1 in 5 people with 2 or more cancer diagnoses have an HCS.^22^ Documentation of family history of cancer was sparse as well, making it more likely that oncologists would miss an opportunity to identify someone who should have been identified as having an HCS at first cancer diagnosis.

### Limitations

This study has some limitations. One was the difficulty in gathering a comprehensive family history from the OMCC’s EHR despite the availability of a family history questionnaire or a pedigree tool. Because family history was typically documented at initial consultation visits, details such as colon polyp history, type of cancer, or age at diagnosis were not updated in follow-up notes or not added at all. Lack of standardization in the documentation of family history information in the EHR is not unique to the OMCC. These findings align with a recent study analyzing the location of family history information in the EHR across the University of Washington Medicine system^23^ that would guide cancer prevention efforts. Historically, no family members’ records were included in patients’ records due to privacy and confidentiality issues, even when it was clinically relevant for cancer treatment or for medical necessity. In 2018, Health Insurance Portability and Accountability Act standards were updated to allow for the inclusion of family records in patients’ EHRs.^24^

The OMCC partners with a surgical pathology team in Port Angeles, Washington. Many biopsies or surgical specimens are sent to an outside laboratory, and pathology reports are scanned back into medical records as PDF documents. Information such as Gleason score, immunohistochemistry for mismatch repair proteins, or pathologic TNM stage had to be manually copied into clinician notes for this information to carry over. These missing details can delay a referral to the genetics service or even prevent ordering testing when a patient has a higher pretest likelihood for an HCS or when NCCN criteria are met. Genetic testing is ordered on many different web portals that are not digitally integrated within the EHR, adding yet another step for clinical staff. This possibly explains why every genetic testing report scanned in the EHR was not added to the patient information faxed for review.

Electronic health record systems were not built to integrate a comprehensive family history of cancer, molecular tumor profiling results, and hereditary genetic testing results. Pedigree tools exist in the EHR, they are not user friendly, and they do not automatically populate the information in a visual format that would raise the suspicion of an HCS. Despite periodic upgrades, EHR systems have not yet kept up with supporting medical oncologists who need an increasing amount of sophisticated data for their day-to-day practice. The challenges we faced during our study highlight the growing need for standardized collection and storage of family history information, tumor profiling data, and hereditary genetic results in a single shared location to maximize the potential benefits of the advances made in cancer care regardless of where the patient is receiving care. Increasing the visibility of patients with cancer who meet NCCN criteria for hereditary genetic testing will also help patients and at-risk family members implement proactive strategies to detect cancer early or prevent it when possible.

Additionally, this study overlapped with the initial major outbreaks of the COVID-19 pandemic. Our respective oncology teams were stretched for time caring for patients with COVID-19, and many steps in our collaboration were delayed. Although we were not able to meet with the OMCC oncology team in person, we were able to establish recurring virtual meetings throughout the course of this study for regular communication relating to clinic processes and to review patient cases together. We envision that remote cancer genetic consultations will be added to multidisciplinary tumor boards as a way to deliver genetic services to patients with cancer in rural and underserved areas.

## Conclusions

In this quality improvement study, there was an increase in the uptake of germline genetic testing by OMCC oncologists for their patients with cancer, even if the measured difference did not reach statistical significance. Efforts to standardize gathering personal and family histories of cancer, review biomarker data suggestive of an HCS, develop workflows to facilitate ordering tumor profiling and/or germline genetic testing every time NCCN criteria are met, advocate for universal coverage for genetic testing, and open data sharing between institutions will help realize the potential benefits of precision oncology for patients and their families seeking cancer care in rural and underserved areas.

## Notes

Supplement.
Data Sharing Statement
