# Supplementary material for: Assessment of a Peer Physician Coaching Partnership Between a Designated Cancer Center Genetics Service and a Community Cancer Network Hospital
Source: JAMA Netw Open. 2023 Mar 6;6(3):e231723. doi: 10.1001/jamanetworkopen.2023.1723 (PMC9989894; doi:10.1001/jamanetworkopen.2023.1723)
Supplement: Supplement. — Data Sharing Statement [file jamanetwopen-e231723-s001.pdf]

Data Sharing Statement

Santos. Assessment of a Peer Physician Coaching Partnership Between a Designated Cancer Center Genetics Service and a Community Cancer Network Hospital. *JAMA Netw Open*. Published March 06, 2023. doi:10.1001/jamanetworkopen.2023.1723

Data
Data available: No

Additional Information
Explanation for why data not available: researchers can email corresponding authors regarding potential collaboration
